# Single-cell RNA sequencing characterization of Holstein cattle blood and milk immune cells during a chronic *Staphylococcus aureus* mastitis infection

**DOI:** 10.1038/s41598-025-96657-5

**Published:** 2025-04-12

**Authors:** Jayne E. Wiarda, Kaitlyn M. Sarlo Davila, Julian M. Trachsel, Crystal L. Loving, Paola Boggiatto, John D. Lippolis, Ellie J. Putz

**Affiliations:** 1 Virus and Prion Research Unit, National Animal Disease Center, Agricultural Research Service, United States Department of Agriculture, Ames, IA USA; 2 Ruminant Diseases and Immunology Research Unit, National Animal Disease Center, Agricultural Research Service, United States Department of Agriculture, Ames, IA USA; 3 Food Safety and Enteric Pathogens Research Unit, National Animal Disease Center, Agricultural Research Service, United States Department of Agriculture, Ames, IA USA; 4 Infectious Bacterial Diseases Research Unit, National Animal Disease Center, Agricultural Research Service, United States Department of Agriculture, Ames, IA USA

**Keywords:** Single-cell, Mastitis, *Staphylococcus aureus*, Chronic, Neutrophil, Granulocyte, scRNA-seq, Infectious diseases, Immunology, Innate immune cells, Granulocytes

## Abstract

Mastitis remains the most prevalent and costly disease to dairy producers. Granulocytes are the primary host innate immune cell responders during infectious mastitis. Here we examine three mid-lactation Holsteins challenged with ~ 150 CFU of *Staphylococcus aureus* (Newbould) that developed chronic mastitis as assessed by bacteria and somatic cell counts in a single quarter. Single-cell RNA-sequencing (scRNA-seq) of blood and milk cells identified immune cell populations of interest from both tissues, and the proportion of cell types recovered via scRNA-seq were highly similar to those recovered via flow cytometry. Granulocytes were the predominating cell type in both blood and milk samples; however granulocytes identified via scRNA-seq revealed several clusters comprised primarily of milk-derived cells. Milk-enriched granulocyte clusters were further investigated to identify gene signatures indicative of the granulocyte-specific localized immune responses in the mammary gland during chronic mastitis infection. Biological process enrichment analysis of gene signatures further revealed relevant networks such as granulocyte migration, myeloid cell differentiation, and inflammatory responses. In total, the work describes the immune landscape occurring at both peripheral and local sites of cattle with mastitis and identified important granulocyte-specific features of the localized immune response occurring during chronic infection.

**Supplementary Information:**

The online version contains supplementary material available at 10.1038/s41598-025-96657-5.

## Introduction

Mastitis continues to plague the dairy industry producers both in veterinary costs and production losses. In the United States alone, it is estimated that mastitis costs the industry ~$2 billion dollars per year^1^. Mastitis is predominantly caused by a bacterial infection of the mammary gland. One of the most common mastitis-causing pathogens is *Staphylococcus aureus (S. aureus)*, which can be associated with treatment resistance and low cure rates^2,3^.

Granulocytes (primarily neutrophils) typically represent the initial immune cell to respond to mastitis infection. High somatic cell counts due to mastitis include immune cells dominated by neutrophils, which migrate to the mammary gland following activation of inflammatory signals and further contribute toward immune recognition and clearance of bacteria. Within the mammary gland, neutrophils generate cytokines, phagocytose bacteria, and produce antimicrobial neutrophil extracellular traps (NETs), which collectively contribute to pathogen control and cell signaling^4–7^.

The specialized role of neutrophils in the mammary gland during infection emphasizes the importance of investigating granulocytes within their respective environments. Single-cell RNA-sequencing (scRNA-seq) has become a powerful tool for characterizing immune cell profiles. scRNA-seq of bovine cells has been reported for peripheral blood mononuclear cells (PBMC)^8,9^, mesenteric lymph node^10^, rumen epithelium^11^, placenta^12^, and ovary^13^ among others. Notably, work has been done investigating bovine peripheral immune cell responses to lipopolysaccharide (LPS) stimulation, but PBMC preparations do not facilitate isolation and thus investigation of granulocytes^9^. Milk cells specifically have also been investigated^14,15^. The initial scRNA-seq evaluation of bovine cells compared a broad suspension of cells from milk and cultured primary bovine mammary epithelial cells (pbMEC)^15^, the latter which typically represent a low percentage of somatic cell populations^16^. Both milk cell suspension and pbMEC samples from a single healthy cow identified 14 cell types including identifiable macrophage, dendritic cell, B, T, and NK cell clusters; however, granulocyte analyses are absent^15^. Recent work evaluating somatic cells from two healthy, mid-lactation Holsteins identified 21 cell types including three neutrophil-specific clusters^14^. While understanding the healthy immune cell repertoire is valuable, it is well established that circulating leukocytes, especially granulocytes, migrate to and infiltrate sites of infection^17–19^, thus vastly altering cellular functions and transcriptomic profiles.

To develop novel mastitis prevention and treatment technologies, understanding the immune profile of infection-fighting cells is critical in both the peripheral (i.e. blood) and localized (i.e. mammary gland) environments. In order to better define immune landscapes occurring at both peripheral and local sites during mastitis infection, we utilized an experimental chronic *S. aureus* challenge (Newbould 305) and scRNA-seq to investigate immune cell profiles in blood and mammary gland during a chronic mastitis infection. We focus on transcriptional dynamics of granulocytes in milk and blood, as these cells have gone understudied in previous scRNA-seq works but play pivotal roles as immune responders to mastitis infection at both peripheral and local sites.

## Results

All cows developed chronic mastitis infections, confirmed by bacterial plating and somatic cell counts (SCC), following *S. aureus* challenge with strain Newbould 305. Post-infection, animals averaged 2.82 × 10^6^ ± 1.08 × 10^6^ somatic cells/mL of the infected quarter. Only infected animals generated sufficient SCC for cell characterization as the NADC healthy herd SCC average was 2.1 × 10^4^ somatic cells/mL. Leukocyte cells from blood and milk samples were collected and analyzed via scRNA-seq, resulting in cell clusters of all major immune cell types.

**Fig. 1:**
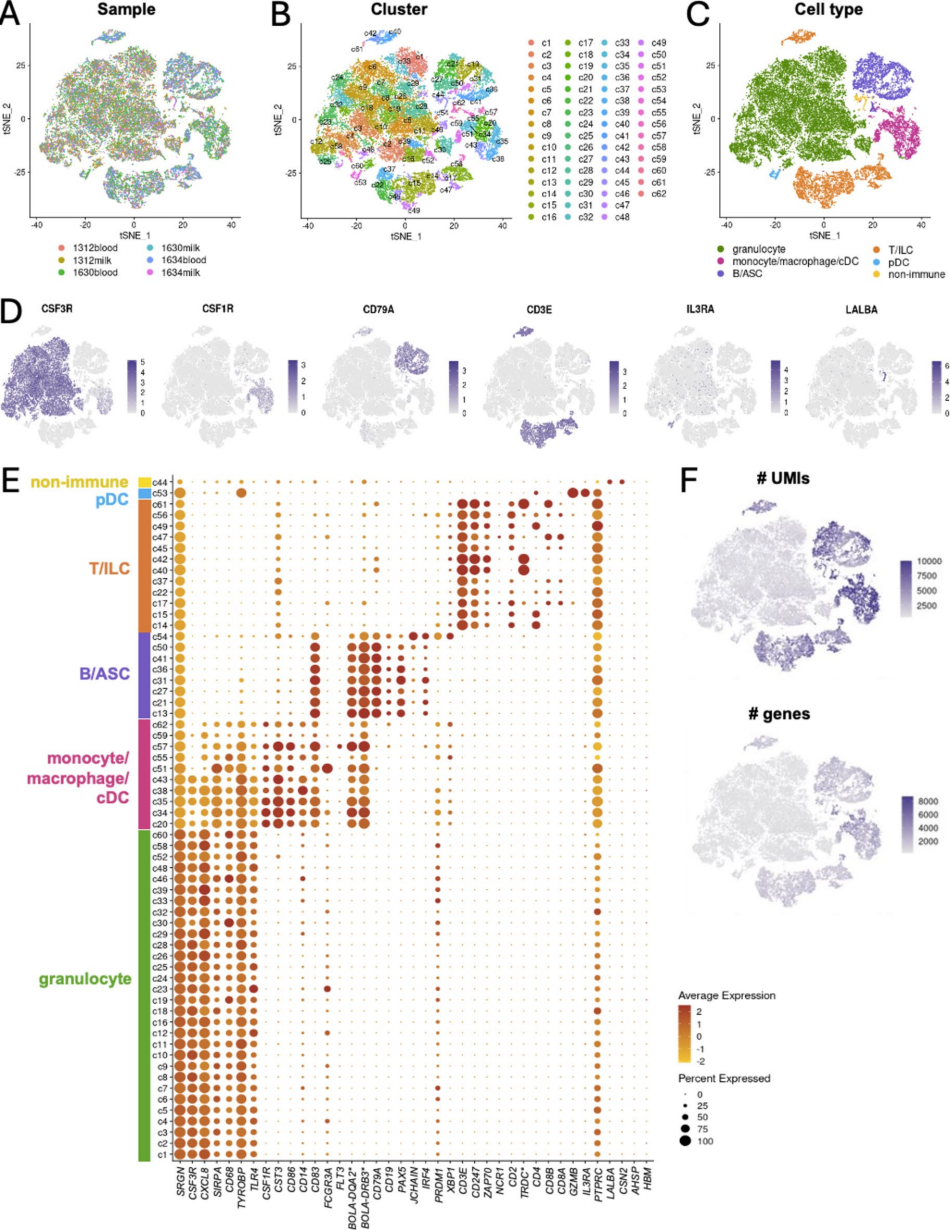
Single-cell RNA sequencing of milk and blood cells from cattle with chronic mastitis reveals six major cell types. (**A**–**C**) t-SNE plots showing sample origin (**A**), cluster assignment (**B**), and cell type annotation (**C**) of cells recovered via scRNA-seq. (**D**) t-SNE plots showing expression of a subset of canonical genes used to annotate cell types shown in (**C**). (**E**) Dot plot of canonical genes (x-axis) used to annotate clusters into cell types (y-axis). Dot size within the plot indicates the percentage of cells in a cluster expressing a gene. Dot fill color indicates the relative expression level of a gene in a cluster. The color bar on the left of the y-axis indicates cell type assignment of clusters. (**F**) t-SNE plots showing number of UMIs (top) or number of genes (bottom) expressed in each cell. In all t-SNE plots (**A**–**D**,**F**), each dot in the plot corresponds to an individual cell. Color of each dot in a plot corresponds to assignment of a cell to sample origin (**A**), cluster assignment (**B**), cell type annotation (**C**), level of gene expression (**D**), or number of UMIs or genes recovered from a cell (**F**). Proximity of cells within a plot is not necessarily a correlate of relatedness. *Gene name is not listed in the genome annotation file but was identified through manual query. See methods section “Gene name replacement” for further information. *ASC* antibody-secreting cell, *cDC* conventional dendritic cell, *ILC* innate lymphoid cell, *pDC* plasmacytoid dendritic cell, *scRNA-seq* single-cell RNA sequencing, *t-SNE* t-distributed stochastic neighbor embedding, *UMI* unique molecular identifier.

In total, 35,338 cells were recovered via scRNA-seq of milk (20,233 total cells) and blood (15,105 total cells) samples collected from each of three Holstein cattle with chronic mastitis (6 samples total; Fig. 1A). Sixty two (62) cell clusters with distinct transcriptional profiles were resolved (see methods; Fig. 1B) with identification of > 4.3 million differentially expressed genes recovered from all pairwise cluster comparisons (Supplementary Fig. 1). Despite their transcriptional diversity amongst the 60 + cell clusters, clusters were assigned to one of six major immune cell types (Fig. 1C) based on conserved, cell type-specific gene expression profiles, including (1) granulocytes (22,020 cells); (2) monocyte/macrophage/conventional dendritic cells (cDCs; (3,187 cells); (3) B cells and antibody-secreting cells (ASCs; (4,076 cells); (4) T cells and innate lymphoid cells (ILCs; (5,473 cells); (5) plasmacytoid dendritic cells (pDCs; (260 cells); and (6) non-immune cells (322 cells). The conserved canonical genes used to assign cell type identities were investigated through general assessment of expression (Fig. 1D,E) and query of genes differentially expressed in each cluster (Supplementary Table 1) to assign cell type identities as described below.

Granulocytes and monocytes/macrophages/cDCs shared expression of many myeloid lineage genes, including *CSF3R*, *CXCL8*, *SRGN*,* TYROBP*,* SIRPA*,* CD68*,* TLR4*,* ADGRE1*, and *HCK*^9,15,20–22^. However, granulocytes lacked expression of other genes used as key markers of monocytes/macrophages/cDCs, including *CD14*,* TREM2*, *AIF1*,* CSF1R*,* CST3*,* CD86*,* CD83*,* FCGR3A*,* FLT3*,* IRF8*,* BOLA-DQA2*and *BOLA-DRB3*^9,15,20,21^. *BOLA-DQA2* and *BOLA-DRB3* were not listed in the genome annotation file but were identified through manual query (see methods section “Gene name replacement” for further information). Moreover, granulocyte clusters had lower transcriptional complexity in terms of both UMIs captured per cell and genes captured per cell, similar to previous reports (Fig. 1F)^23^. B cells and ASCs expressed genes encoding for components associated with B cell receptor (BCR) signaling (*CD79A*,* CD79B*,* CD19*,* MS4A1*,* BLNK*), antibody secretion (*JCHAIN*), and B lineage-defining transcription factors (*PAX5*, *IRF4*)^9,15,20,21,24^. T cells and ILCs were annotated based on expression of genes encoding for proteins associated with T cell receptor (TCR) complex signaling (*CD3E*,* CD3D*,* CD3G*,* CD247*,* ZAP70*), T cell subset markers (TRDC*, *CD4*,* CD8A*,* CD8B*), and markers characteristically expressed by natural killer (NK) cells, other ILC subsets, and/or cytotoxic T cells (*NCR1*,* KLRB1*,* NKG2A*,* KLRD1*,* NKG7*,* GNLY*,* CTSW*,* PRF1*)^9,15,20,21^. pDCs also expressed *CD4* and further expressed additional pDC marker genes, including *GZMB* and *IL3RA*^25^. Non-immune cells were identified due to high expression of *LALBA* and *CSN2*, markers characteristic of bovine mammary epithelial cells^15^. Non immune cells also had very few cells expressing *PTPRC* (encoding CD45), a pan-leukocyte marker typically expressed by immune cells recovered via scRNA-seq^20^. Genes characteristic of erythrocytes were also queried but found to be absent (*AHSP*,* HBB*,* HBM*)^21^, indicating little to no red blood cell contamination in the dataset.

**Fig. 2:**
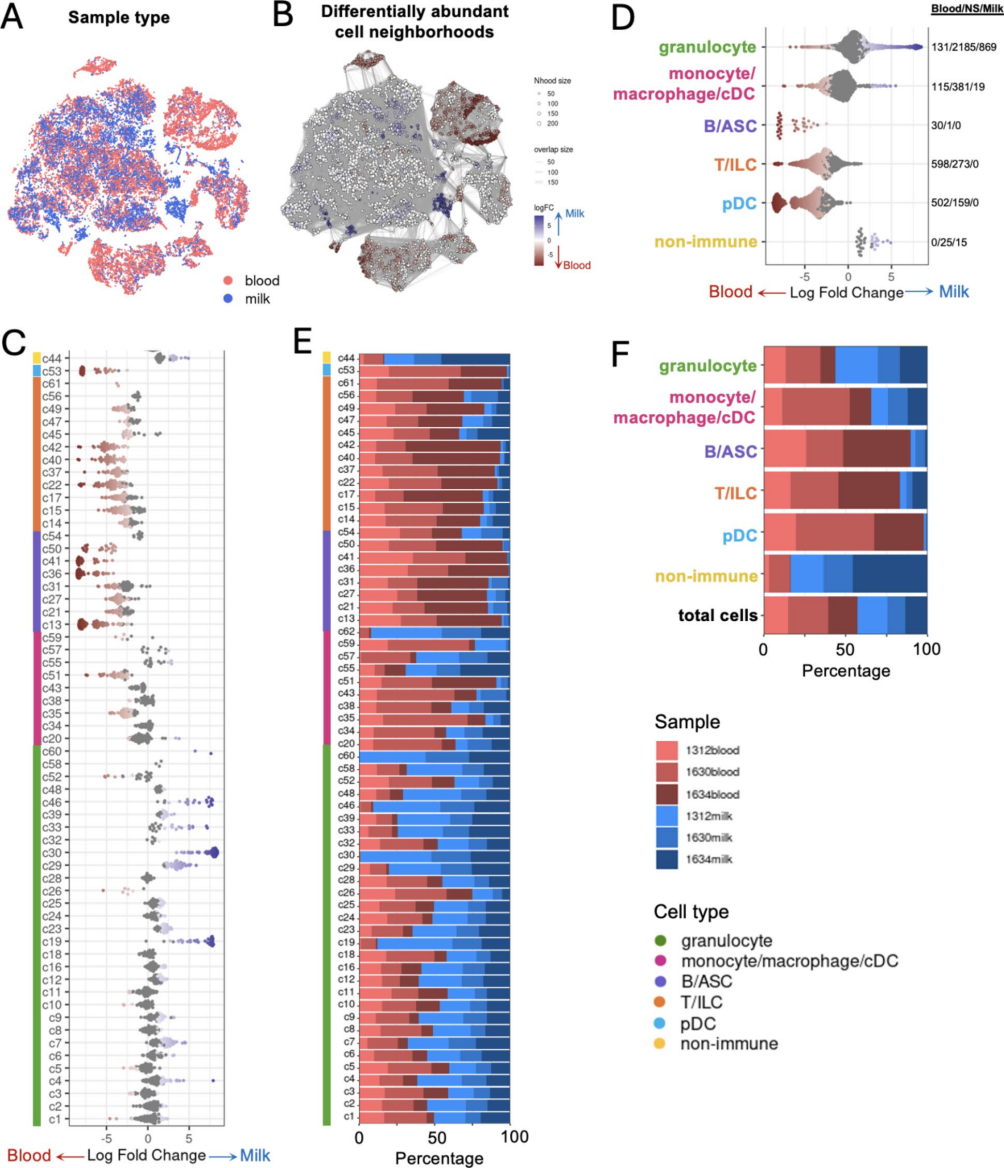
Cell types exhibit different abundances in milk versus blood samples recovered from cattle with chronic mastitis. (**A**) t-SNE plot showing sample type cells were recovered from for scRNA-seq. Each dot in the plot corresponds to an individual cell. Color of each dot in the plot corresponds to assignment of a cell to milk (blue) or blood (red) samples. Proximity of cells within a plot is not necessarily a correlate of relatedness. (**B**) Results of differential abundance testing for cell neighborhoods overlaid onto t-SNE coordinates shown in (**A**). Each dot in the plot represents one cell neighborhood. Size of the dot corresponds to the number of cells in a neighborhood. Width of lines connecting neighborhoods corresponds to the number of cells shared between neighborhoods. The color of a neighborhood corresponds to significantly greater abundance in blood (red), milk (blue), or no significant difference in abundance (grey). Shade of red (blood) or blue (milk) fill in cell neighborhoods corresponds to the logFC in abundance. A neighborhood was considered differentially abundant if the spatially-corrected p-value was < 0.05. (**C**,**E**) Beeswarm plots of differential abundance results shown in B separated for individual cell clusters (**C**) or cell types (**E**) (y-axes). A neighborhood was assigned to a cluster (**C**) or cell type (**E**) if > 90% of cells in a neighborhood belonged to a single cluster. Differential abundance testing results for neighborhoods with ≤90% of cells recovered from a single cluster (**C**) or cell type (**E**) are not shown. Each dot in the plot represents one cell neighborhood. The color of a dot corresponds to significantly greater abundance in blood (red), milk (blue), or no significant difference in abundance (grey). Position on the x-axes and shade of red (blood) or blue (milk) fill in dots corresponds to the logFC difference in abundance. In C, the color bar to the left of the plot corresponds to the cell type each cluster was assigned to. To the right of each plot, the number of neighborhoods exhibiting significantly higher abundance in blood, no significant difference in abundance, and significantly higher abundance in milk are sequentially listed for each cluster (**C**) or cell type (**F**). A neighborhood was considered differentially abundant if the spatially-corrected p-value was < 0.05. (**D**,**F**) Stacked bar plots showing the proportion of cells (x-axes) comprising each cluster (**D**) or cell type (**F**) (y-axes). Fill color of bars corresponds to sample cells were derived from. In (**D**), the color bar to the left of the plot corresponds to the cell type each cluster was assigned to. Bar size is proportional to 100% of cells comprising a cluster (**D**) or cell type (**F**) and is not proportional to the total absolute number of cells in each group. *ASC* antibody-secreting cell, *cDC* conventional dendritic cell, *ILC* innate lymphoid cell, *logFC* log fold change, *Nhood* neighborhood, *NS* not significant, *pDC* plasmacytoid dendritic cell, *scRNA-seq* single-cell RNA sequencing, *t-SNE* t-distributed stochastic neighbor embedding.

Milk somatic cells are dominated by granulocytes, most-so during infectious states where neutrophils undergo diapedesis to enter the mammary gland from the blood^4^ leading to vastly different cell profiles between blood and milk samples. Therefore, we initially investigated the differences in cell compositions recovered via scRNA-seq from milk and blood samples of cattle with chronic mastitis (Fig. 2A). Cells with high degrees of transcriptional similarities were allocated into cell neighborhoods, and within each cell neighborhood, the sample origin of each cell was determined and used to test for statistical differences in abundance for milk versus blood samples. Differential abundance testing resulted in identification of cell neighborhoods that were significantly enriched in milk or blood samples as shown in Fig. 2B. Cell neighborhoods were further assigned back to cell annotations at the cluster (Fig. 2C) or cell type (Fig. 2D) level. Within a cell cluster, significant differential abundance was exhibited in at least one cell neighborhood, with the exception of c18, c28, c34, c43, c56, and c58 that did not have any neighborhoods with significant differential abundance detected (Fig. 2C). Cluster-level findings indicated neighborhoods exhibited significantly greater abundance in blood for B/ASC, T/ILC, and pDC clusters, significantly greater abundance in milk for non-immune clusters, and a mixture of significantly greater abundance in milk versus blood for granulocytes and monocyte/macrophage/cDCs (Fig. 2C). Figure 2D summarizes the previously made observations at the cell type level, again showing significant differential abundance biased towards blood in B/ASCs, T/ILCs, and pDCs; biased towards milk in non-immune cells; and biased towards both milk and blood for granulocytes and monocyte/macrophage/cDCs. Overall, these findings could also be generally observed in the cell compositions obtained from each cluster or cell type, as shown in Fig. 2E and F, respectively.

**Fig. 3:**
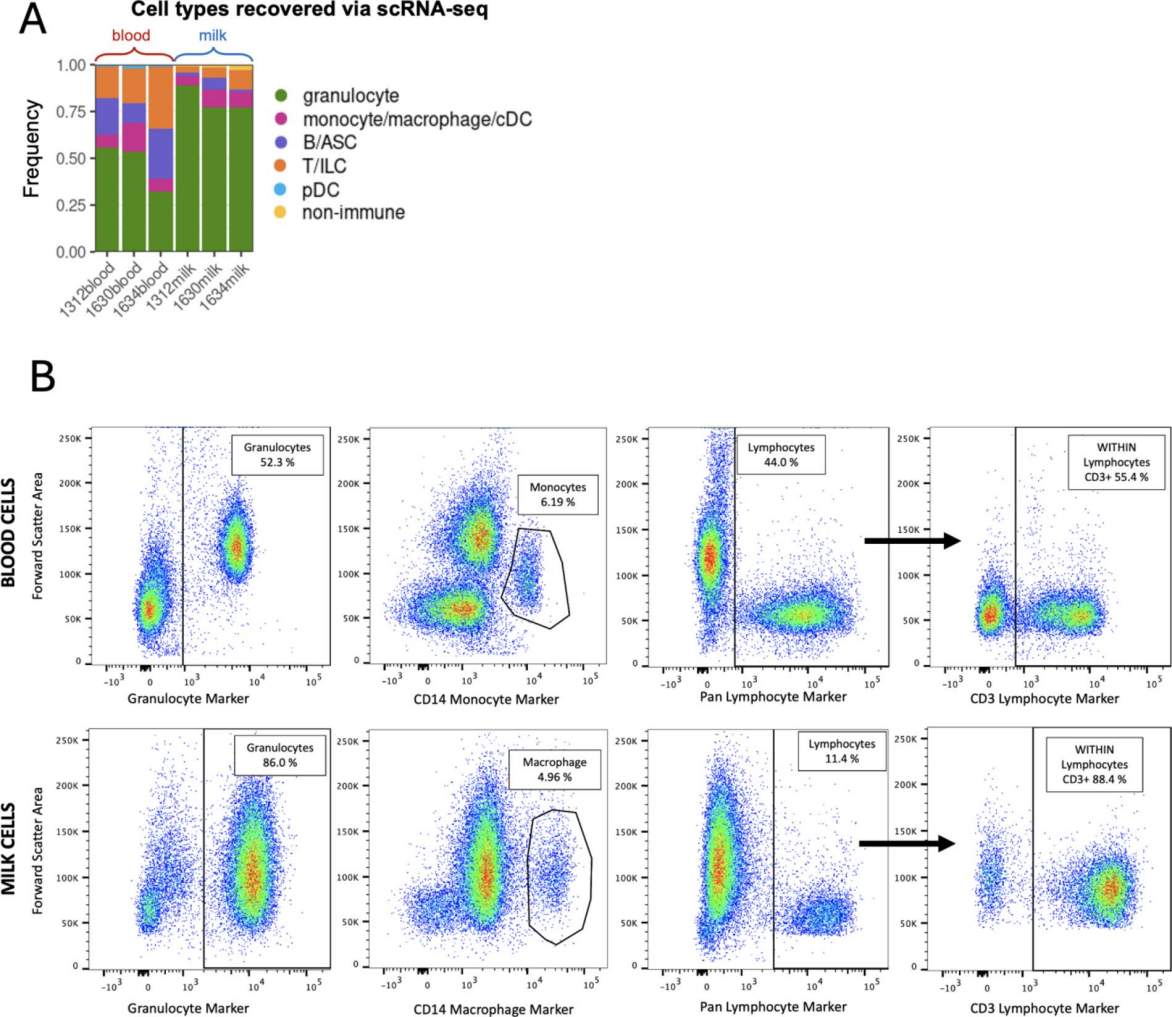
Similar cell type proportions recovered from milk and blood via single-cell RNA sequencing and flow cytometry. (**A**) Stacked bar plot showing the proportion of cells (y-axis) comprising each sample used for scRNA-seq (x-axis). Fill color of bars corresponds to cell type. Bar size is proportional to 100% of cells comprising a sample and is not proportional to the total absolute number of cells in each sample. (**B**) Comparative cell type percentages as indicated by flow cytometry analysis. Percentages are relative to CD45 + live single cells with the exception of CD3 + cells whose percentages are within CD45 + live single cell lymphocytes (black arrow). *ASC* antibody-secreting cell, *cDC* conventional dendritic cell, *ILC* innate lymphoid cell, *logFC* log fold change, *pDC* plasmacytoid dendritic cell, *scRNA-seq* single-cell RNA sequencing.

Cell compositions for general cell types obtained via scRNA-seq between milk and blood samples were further validated by flow cytometry experiments conducted on milk and blood samples. Monocyte/macrophage, combined lymphocyte, B, T/ILC populations, and granulocyte cell type proportions determined through scRNA-seq analysis (Fig. 3A) were highly similar to those identified by flow cytometry (Fig. 3B). As reported previously, milk cells from infected quarters were dominated by granulocytes^26^, but populations of macrophages (cells expressing CD14), lymphocytes, and T cells (lymphocytes expressing CD3) were also present in similar proportions between scRNA-seq and flow cytometry data of milk.

**Fig. 4:**
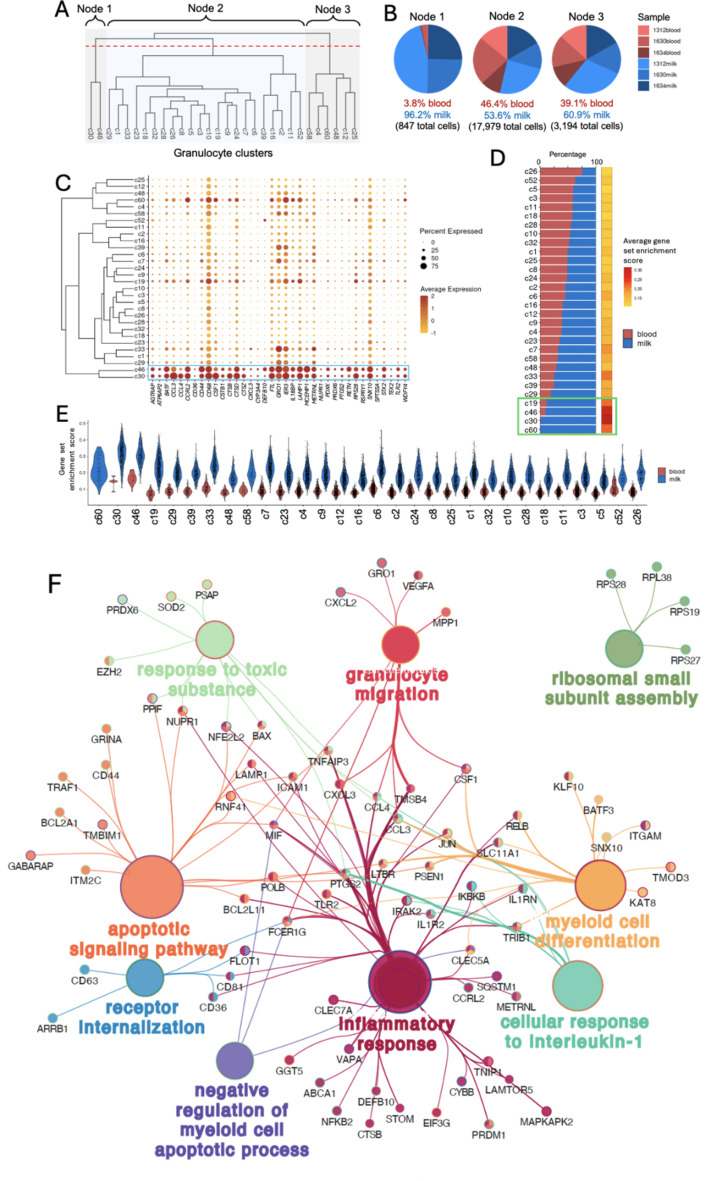
A milk-enriched granulocyte gene signature indicates transcriptional dyanmics of the granulocyte-specific, localized immune response to mastitis. (**A**) Phylogenetic tree indicating the transcriptional relatedness of granulocyte clusters recovered via scRNA-seq. The red dotted line indicates high-level segregation of hierarchical clustering into nodes referenced in the results. (**B**) Pie charts showing the proportion of cells (pie slices) comprising the cells within each node identified in (**A**). Fill color of pie slices corresponds to sample identifiers. Pie size is proportional to 100% of cells comprising a node and is not proportional to the total absolute number of cells in each node. (**C**) Dot plot of signature genes (x-axis) and their expression patterns across granulocyte clusters (y-axis). Signature genes were identified for node 1 identified in (**A**). The full node 1 gene set (210 genes) is not shown and can instead be found in Supplementary Fig. 2. Only genes with log2FC > 1 for both clusters (c30, c46) are shown in this figure. Dot size within the plot indicates the percentage of cells in a cluster expressing a gene. Dot fill color indicates the relative expression level of a gene in a cluster. The phylogenetic tree on the left shows relatedness of granulocyte clusters, identical to as shown in (**A**). (**D**) Left: Stacked bar plot showing the percentage of cells (x-axis) comprising each granulocyte cluster used for scRNA-seq (y-axis). Fill color of bars corresponds to cells derived from blood (red) or milk (blue) samples. Bar size is proportional to 100% of cells comprising a cluster and is not proportional to the total absolute number of cells in each cluster. Clusters on the y-axis are listed (top to bottom) in ascending order of the percentage of milk cells comprising each cluster. Right: heatmap showing the average gene set enrichment score for the 210-gene node 1 gene signature. Fill color corresponds to the average gene set enrichment score for each granulocyte cluster. (**E**) Violin plots showing gene set enrichment scores (y-axis) for the 210-gene node 1 gene signature. Granulocyte clusters (x-axis) are separated into cells derived from blood samples (red) or milk samples (blue). c60 does not have a violin plotted for blood since no blood-derived cells were identified in the cluster. Individual points within each violin represent single cells. (**F**) Cytoscape Gene Ontology (GO) term enrichment and clustering analysis of the 210-gene node 1 gene signature. *scRNA-seq* single-cell RNA sequencing.

Of exceptional interest in the context of mastitis infection were differentially expressed genes amongst granulocyte clusters (total of 8981 DEGs detailed in Supplemental Table 2). Further investigation was conducted to uncover potential gene signatures that may be unique to milk samples and thus indicative of a granulocyte-specific, localized immune response to mastitis. Hierarchical clustering of all granulocyte clusters revealed early transcriptional divergence of c30 and c46 into a separate node at a high level of classification (Fig. 4A). Assessment of the cellular compositions of high-level nodes revealed Node 1 (containing c30 and c46) was almost exclusively comprised of milk-derived cells (96.2%), while other nodes (Node 2, Node 3) contained a mixture of both milk and blood cells (Fig. 4B). A Node 1-enriched gene signature was created by identifying all genes with significantly greater expression in both c30 and c46 relative to other granulocyte clusters, yielding a list of 210 genes (Fig. 4C, blue box & Figure S2). Gene set enrichment analysis of the 210-gene signature revealed granulocyte clusters comprised of higher percentages of milk cells generally had higher average enrichment scores for the gene signature (Fig. 4D). In fact, the top four clusters with the highest percentage of milk cells (> 88% per cluster) also had the four highest average gene set enrichment scores (Fig. 4D, green box). Closer dissection of cells derived from milk versus blood samples within each granulocyte cluster demonstrated higher enrichment of the Node 1 gene signature for milk-derived cells compared to blood-derived cells across every granulocyte cluster, indicating the 210-gene signature could be ubiquitously applied to granulocyte clusters as a milk-enriched transcriptional program (Fig. 4E). Thus, the 210-gene milk-enriched granulocyte signature serves as an indicator of granulocyte-specific transcriptional programming of the localized immune response to mastitis. To determine functional implications of the milk-enriched granulocyte transcriptional program, the 210-gene signature was used as input for identification of enriched Gene Ontology (GO). The gene signature was significantly enriched for 65 biological process GO terms that clustered into 9 functional groups (Fig. 4F), the details of which are available in Supplemental Tables 3, and include terms such as ‘innate immune response’, ‘granulocyte chemotaxis’, ‘response to bacterium’, ‘toll-like receptor signaling pathway’, and ‘innate immune response-activating signal transduction’.

## Discussion

The requirements of calving and lactation, milk harvest, and general care of dairy cows results in unavoidable exposure to a plethora of bacterial pathogens; thus making the complete eradication of mastitis unlikely. However, improved understanding of mechanisms of disease and host immune response, especially in the localized mammary gland environment, are important tools in the development of novel and improved prevention and treatment strategies. Clinical signs of mastitis can include increased somatic cell counts, inflammation of the udder, reduced milk quality and milk production, as well as secretory cell damage, all of which can contribute to cows being culled from the herd^27,28^. *Staphylococcus aureus* remains one of the most common mastitis causing pathogens, and unlike some acute bacterial infections, *S. aureus* can result in chronic infections, with consistently detectable increased SCC and bacterial burden^3,26,29^. Here for the first time, we utilize constantly advancing scRNA-seq technologies and a chronic *S. aureus* model to investigate the immune cell profile of blood and milk during mastitis.

Our data set resulted in 62 identifiable cell type clusters across blood and milk samples and clearly identified six major cells types; granulocytes, monocyte/macrophage/conventional dendritic cells, B cells and antibody-secreting cells, T cells and innate lymphoid cells, plasmacytoid dendritic cells, and non-immune cells. While all primary cell types were identified in both the peripheral blood and mammary gland, certain clusters were over represented in either blood or milk, and major cell types were unevenly distributed between environments. Granulocytes dominated the largest proportion of analyzed milk cells, while blood contained substantially more lymphocytes; these findings were further validated by flow cytometry. The capture of so many granulocyte specific clusters in both blood and milk is a highlight of this work as obstacles in neutrophil adaptation to scRNA-seq analysis include lower RNA content, cell viability sensitivity, and protease/nuclease activity. Collectively, these have limited milk cell and even human cell scRNA-seq characterization of granulocytes^15,30^. Characterization of granulocyte differences across environments may facilitate novel mastitis treatment and/or prevention strategies by targeting localized or systemic responses. For example, it may be possible to develop methodology to increase production of innate immune cell progenitors, enhance migration of circulating granulocytes to the mammary gland, and/or activate milk specific granulocyte populations.

First lines of cellular immune defense for bacterial infection emphasizes attention to granulocytes, primarily neutrophils, which are a focus of mastitis research. Granulocytes are a highly heterogenous cell population whose transcriptional profiles are sensitive to activation signals, pathogen defense, and maturation processes^30–32^. Across all granulocyte clusters, there were 8981 DEGs indicating components of each of these elements (see Supplementary Table 2). Some examples include the IL-8 receptor, *CXCR2*, which was differentially expressed (DE). Polymorphisms of this gene have been associated with incidence of mastitis and as well as survival, migration, and pathogen killing by granulocytes^33^. Transcription factors such as *IRF7* were significantly differentiated amongst clusters, which has been associated with human patients experiencing mild versus severe disease and is heavily implicated in neutrophil cytokine production^31^. DEGs further included *ITGAM*, *VIM*, and *MMP9*, which are genes associated with immature neutrophils signatures, as well as activated interferon inducible neutrophil subsets with differential expression among *IFIT*(1 & 2), *IFITM* (2 & 3), and *ISG15* genes^30,32^.

Another notable feature of this work is the characterization of granulocyte activity within the mammary gland as well as the periphery. Within the 30 granulocyte clusters identified within our study, unequal representation in blood and milk was evident. This disease model allows for a localized immune response to the mammary gland, thus facilitating the unique identification of a milk-based granulocyte signature. Hierarchical clustering established three separate nodes of related gene signatures and revealed that the highest enrichment was encountered in milk dominated granulocyte clusters, particularly c30 and c46, which share significantly higher expression of 210 genes. Those with the greatest enrichment included *CXCL3* which is a primary neutrophil chemotaxin^34^, the classic Toll-like Receptor 2 (*TLR2*) associated with response to Gram-positive bacteria^35^, and CD36 which is implicated in neutrophil recruitment, cytokine production, phagocytosis, and macrophage clearance of compromised granulocytes^36^. Gene Ontology (GO) enrichment further illustrated this milk based granulocyte signature with clustering terms including ‘granulocyte migration’, ‘inflammatory response’, ‘apoptotic signaling pathway’, and ‘myeloid cell differentiation’ which suggests that granulocytes localized to the mammary gland are undergoing multi-faceted components of activation, migration, and functional immune response to the chronic, yet localized infection.

Limitations of this study include a lack of scRNA-seq data for control non-infected milk cells. Although our research herd SCC were too low for suitable cell isolation and sequencing this will be an important emphasis for future objectives and comparisons. This study also only examines cell populations from a chronic experimental infection. Studies of acute infections from additional pathogens and co-infections would be beneficial to the dairy industry, both from experimental and field collection instances. Nonetheless, to our knowledge this is the first characterization of granulocytes from milk using scRNA-seq, which represents an important step in utilizing this robust methodology to inform mastitis treatment and prevention.

Host immune responses to naturally occurring mastitis infections can vary wildly depending on environment, stage of lactation, physical fitness, virulence of pathogen, and additional factors. The range of causative pathogens and complexity of mastitis infections has limited the treatment and prevention strategies available to producers. Commercial products targeted toward prophylactic mastitis treatment have previously failed, possibly in part to incomplete bacterial defense innate immune activation. The heterogeneity and pathogen-specific profiles of activated/anti-bacterial granulocytes explored at the scRNA-seq level represents the next frontier of mastitis research. Characterization of localized mammary specific granulocytes at the single cell level have the capacity to fill knowledge gaps for granulocytes and other immune cells. Future scRNA-seq work may facilitate understanding of disease pathogenesis and development of granulocyte targeted prevention and treatment technologies based on novel findings of granulocyte maturation, migration, and/or activation.

## Materials and methods

### Experimental design

From the National Animal Disease Center (NADC) research herd, three multi-parity lactating Holstein cows were utilized. All three animals were approximately four months post-challenge with ~ 150 colony forming units (cfu) *Staphylococcus aureus* (Newbold strain) via intramammary infusion in 2 ml of phosphate buffered saline (PBS) into a single quarter. All cows developed chronic mastitis infection as determined by somatic cell and bacterial counts (described below). The study was conducted in accordance with relevant guidelines and regulations. All protocols and procedures were approved by the NADC Animal Care and Use Committee and performed in accordance with ARRIVE guidelines.

## Bacterial and somatic cell counts

Milk samples were aseptically collected from infected quarters and subsequently diluted and plated for culture on MacConkey agar plate (BD Biosciences, San Jose, CA, USA Cat. No.211387). Following overnight incubation at 37ºC, single colonies were counted and morphology confirmed as previously reported^26^. Bacterial counts averaged 1.62 × 10^7^ ± 8.30 × 10^6^ cfu/mL. Somatic cell count (SCC) samples were collected and submitted to the Dairy Lab Services (Dubuque, IA, USA).

## Milk and blood cell Preparation

For milk cell isolation, 465 mL of milk were collected from the experimentally infected quarter under sterile conditions. Milk was diluted with 35mL of PBS, inverted to mix, and centrifuged at 1500 g for 45 min at 4–6ºC with the brake off. The cream fat layer and skim milk were removed, and pellets were resuspended in 10 mL of PBS. Samples were treated with two rounds of ACK lysis buffer (20 mL, rocking for ~ 60 s, centrifuged at 250x for 10 min). Final cell pellets were resuspended in 10 mL of DMEM supplemented with 10% fetal bovine serum (FBS). For isolation of white blood cells, 54 mL of blood was obtained from the jugular vein and placed into 6 mL 2X acid citrate dextrose (ACD). Total 60 mL blood samples were split in half, each diluted with 20 mL of PBS, and centrifuged at 1500x for 20 min with the brake off. The supernatant containing serum was removed and remaining buffy coat and cell pack were treated with two rounds of ACK lysis buffer (20 mL, rocking for ~ 60 s, centrifuged at 250x for 10 min). Final cell pellets were resuspended in 10 mL of DMEM supplemented with 10% FBS. Both milk and blood cells were then washed twice in supplemented Hank’s Balanced Salt Solution (HBSS; ThermoFisher), 0.5% BSA (Sigma-Aldrich), and 2 mM ethylenediaminetetraacetic acid (EDTA; ThermoFisher)) and viable cells enumerated using the Count and Viability Assay Kit on the MUSE^®^ detection system (Merck Millipore).

### Flow cytometry

For flow cytometry, 3mL of EDTA treated whole blood and 100 mL milk from each experimental quarter were sterilely collected. Milk was centrifuged at 4ºC at 2000×*g* for 40 min, fat and skim layers were removed, and cell pellets were resuspended with complete media (RPMI + L glutamine and 10% FBS). Staining of milk cells and whole blood was completed as reported previously^26^. Briefly, cells were stained with a live-dead dye (1:100 diluted Zombie Yellow BioLegend, San Diego, CA, USA Cat. No. 423103/423104) and independent directly conjugated, primary, and secondary antibodies as detailed in Table 1. Data was acquired using a Becton Dickinson LSR II flow cytometer and analyzed using FlowJo software (FlowJo LLC, Ashland, OR, USA). Both milk and blood cells were gated to analyze only live cells that were CD45^+^. CD14 + cells were labeled as monocytes/macrophages, and granulocytes labeled using the granulocyte marker (Table 1). Lymphocytes were identified using a pan-lymphocyte marker and within the lymphocyte population was evaluated for CD3E^+^ T cells.

**Table 1:** Antibodies utilized to characterize whole blood and isolated milk cells.

Marker	Primary Antibodies	Secondary Antibodies
CD45	Monoclonal Antibody Center, Washington State University, USA. Cat. No. BOV2039	BioLegend, San Diego, CA, USA Brilliant Violet 421™ anti-mouse IgG2a, Cat. No. 407,117
Granulocyte Marker	Monoclonal Antibody Center, Washington State University, USA. Cat. No. BOV2068	BioLegend, San Diego, CA, USA APC/Cy7 anti-mouse IgG1 Antibody, Cat. No. 406,620
CD14	BioLegend, San Diego, CA, USA Cat. No. 301,812	Directly Conjugated
Pan-Lymphocyte	Monoclonal Antibody Center, Washington State University, USA. Cat. No. BOV2071	BD Biosciences, San Jose, CA, USA BUV395 Rat Anti-Mouse IgG3, Cat No. 744,138
CD3	Monoclonal Antibody Center, Washington State University, USA. Cat. No. BOV2009	BD Biosciences San Jose, CA, USA AF350 Goat Anti-Mouse IgG1, Cat No. A21120

### scRNA-seq library Preparation & sequencing

Similar to previous work^20,21^, cells were processed for scRNA-seq using the Chromium Single Cell 3’ Gene Expression kit v3.1 (10X Genomics), targeting capture of 10,000 cells per sample. cDNA libraries were sequenced across 3 lanes on a HiSeq 3000 (Illumina).

### scRNA-seq data processing & analysis

#### Read alignment and counting

A gene count matrix was generated using CellRanger v7.2 (10X Genomics) and the ARS-UCD1.2 *Bos taurus* genome, Ensembl annotation release 107^37^.

#### Ambient RNA removal

Ambient RNA was calculated and removed from samples using the auto-estimation method available from R software package, SoupX v1.6.2^38^ as previously described^20,21,24,39^.

#### Doublet estimation

Doublets were identified using scDblFinder v1.18.0^40^ with default parameters and the ‘clusters’ flag enabled.

#### Data filtering

Cell and gene filtering was performed similar to previous work^20,21,24,39^. Custom R scripts were created to remove cells with doublet classification, > 12.5% mitochondrial genes, < 250 total genes, and/or < 500 total UMIs.

#### Data normalization, integration, dimensionality reduction, scaling, and nearest neighbor calculations

The R software package, Seurat v4.1.1^41^, was used for remaining data analysis, unless otherwise specified. Data from each sample were separately normalized using SCT normalization^42^ with Seurat function *SCTransform()* with default parameters. Samples were integrated using Seurat functions *SelectIntegrationFeatures()*,* PrepSCTIntegration()*,* FindIntegrationAnchors()*, and *IntegrateData()* with default parameters and SCT-normalized data^43^. Principal component analysis was conducted from integrated data using Seurat function *RunPCA()* to calculate principal components (PCs). The first 30 PCs were used to calculate t-distributed stochastic neighbor embedding (t-SNE) coordinates using Seurat function *RunTSNE()* and to calculate nearest neighbors using Seurat function *FindNeighbors()*. Data in the RNA assay were normalized and scaled using Seurat functions *NormalizeData()* and *ScaleData()*, respectively, with default parameters.

#### Cell clustering and annotation

Cell clustering was performed and assessed similar to previous work^20^. Clustering was performed with the Seurat function *FindClusters()* at multiple resolutions, ranging from 0.5 to 10 in increments of 0.5. The R software package clustree v0.5.0^44^ was used to visualize the relationships between clustering resolutions with the function *clustree()*. The smallest clustering resolution (4.0) that could successfully segregate cells of distinct major groups (pDC, monocyte/macrophage, B/ASC, T/ILC, non-immune, granulocytes) was selected for downstream use based on analysis of canonical genes described in results. An exception was made for cluster 53, which was composed of cycling B and myeloid lineage leukocytes and remained as a single cluster at all tested clustering resolutions. A data subset of cluster 53 was created using the Seurat *subset()* function and re-clustered at a resolution of 2.0 to separate cycling B from cycling myeloid lineage cells based on query of canonical gene expression. Enhanced annotation of cluster 53 into cycling B or cycling myeloid cells was then incorporated into clustering results at resolution 4.0 performed on the total dataset to extract a total of 62 clusters used for further analyses. Clusters were renamed as c1 through c62 based on descending size (total number of cells belonging to a cluster). Clusters were further assigned to major cell groupings by assessing canonical gene expression and differential gene expression patterns described in results.

#### Differential gene expression analysis

For differential gene expression analysis of one cell cluster versus all other clusters in the dataset, the Seurat function *FindAllMarkers()* with default parameters was used. For pairwise differential gene expression analysis between clusters, the Seurat function *FindMarkers()* was used. A gene was only considered differentially expressed if expressed in at least 10% of cells in one compared population, a |log2FC| value > 0.25 was obtained, and a corrected p-value < 0.05 was found.

#### Differential abundance analysis

The R software package, miloR v1.4.0^45^ was used to perform differential abundance analysis. Seurat object data were converted to a SingleCellExperiment object (*as.SingleCellExperiment()*)^46^ and then to a milo object (*Milo()*). A graph of nearest neighbors was built from pre-existing neighborhood graph created using Seurat (*graph(buildFromAdjacency())*, specifying k = 20 was used to detect nearest neighbors in Seurat. Cell neighborhoods were created using the function *makeNhoods()*, specifying to use 20% of total cells, a k parameter of 20, 30 PCs, and a refined, graph-based approach. A neighborhood graph was built (*buildNhoodGraph()*), and cells belonging to each sample were counted within each neighborhood (*countCells()*). Differential abundance analysis was performed, using cell origin from milk versus blood samples as the experimental variable within the function *testNhoods()* with graph-overlap as the method used to calculate spatial false discovery rate (FDR) weighting. A corrected spatial FDR value < 0.05 was considered significant. Cell neighborhoods were assigned to cluster identities if > 90% of cells in a neighborhood belonged to a single, respective cluster (*annotateNhoods()*). Cell neighborhoods were assigned to major cell group identities if > 90% of cells in a neighborhood belonged to a single, respective major cell group (*annotateNhoods()*). Cell neighborhoods with < = 90% of cells belonging to a single annotation were classified as mixed cell neighborhoods and were not assessed further.

#### Hierarchical clustering

Hierarchical clustering of the 62 defined clusters or 30 defined granulocyte clusters was performed using the Seurat function *BuildClusterTree()* with the integrated assay and first 30 PCs.

#### Granulocyte data subsetting

A data subset comprised of only cells identified as granulocytes was created similar to previous work^20,24,39^. The *subset()* function was used to select only granulocyte cells, followed by *DietSeurat()* to remove all data transformations and reductions, with the exception of t-SNE coordinates. Data were next reprocessed through data normalization, integration, dimensionality reduction (excluding t-SNE), and scaling as described above.

#### Granulocyte gene signatures

To identify gene signatures for granulocyte populations enriched in milk samples, differential gene expression testing was performed for each granulocyte cluster relative to all other granulocytes (using the *FindAllMarkers()* command described above). Genes differentially expressed and with log2FC > 0.25 for all clusters considered in a gene signature were then identified (i.e. genes differentially expressed and with log2FC > 0.25 for both clusters c30 and c46). Clusters to consider for the milk-enriched signature were identified as a separate node with a high percentage of milk-derived cells recovered via hierarchical clustering of all granulocyte clusters (described above).

#### Gene set enrichment analysis

The milk-enriched granulocyte gene signature obtained as described above (210 genes total) was utilized as a gene set. Gene set enrichment analysis was performed to obtain a gene set enrichment score for each cell annotated as a granulocyte with AUCell v1.18.1^47^ as previously described^21^. Within each individual cell, AUCell utilizes rank-based ordering of raw gene counts followed by calculation of the percentage of genes represented in the top proportion of highest ranked genes (default value of 5% used here) in order to calculate area under the curve (AUC) as a gene set enrichment scoring metric.

#### Gene ontology (GO) analysis

Gene Ontology (GO) term enrichment and clustering analysis were also performed on the 210 genes from the granulocyte gene signature using the ClueGo Plug-in in Cytoscape 3.9.1 software^29^. Biological function GO term enrichment was calculated with a hypergeometric test. Functional grouping was based on the kappa score. Terms were declared as connected with a kappa score greater than 0.4. Redundant groups with greater than 50% overlap were merged. Mapped genes represent at least 4% of the total associated genes per term.

#### Gene name replacement

Some gene names used in-text and in main figures were not listed in the genome annotation file but were identifiable through manual query^48^, similar to gene annotation limitations noted in previous work^21,49^. These gene names are listed with an asterix (*) and correspond to the following Ensembl identifiers found in the annotation file used: *TRDC* = *ENSBTAG00000055197*; *BOLA-DQA2* = *ENSBTAG00000009656*; *BOLA-DRB3* = *ENSBTAG00000013919*.

## Electronic supplementary material

Below is the link to the electronic supplementary material.

Supplementary Material 1

Supplementary Material 2

Supplementary Material 3

Supplementary Material 4

Supplementary Material 5

Supplementary Material 6

## Data Availability

Scripts used for computational analyses of scRNA-seq data are available at https://github.com/SwiVi/scRNAseq_ChronicMastitis_MilkBlood. Raw sequencing data are available for download at SRA under accession number PRJNA1114020 and at the following link: (https://dataview.ncbi.nlm.nih.gov/object/PRJNA1114020?reviewer=k3825rmph0h1vqdfuf3gn6l5u5). Supplementary data and processed scRNA-seq data objects are available for download at Ag Data Commons submission link: (https://agdatacommons.nal.usda.gov/articles/dataset/Single-cell_RNA_sequencing_data_and_resources_from_blood_and_milk_immune_cells_of_Holstein_cattle_with_chronic_mastitis_caused_by_experimental_i_Staphylococcus_aureus_i_infection/26870506/1). Data is available for interactive online query at https://singlecell.broadinstitute.org/single_cell/study/SCP2747/single-cell-rna-sequencing-of-holstein-cattle-blood-and-milk-immune-cells-during-a-chronic-staphylococcus-aureus-mastitis-infection.
