# Supplementary material for: Single-cell RNA sequencing characterization of Holstein cattle blood and milk immune cells during a chronic Staphylococcus aureus mastitis infection
Source: Sci Rep. 2025 Apr 12;15:12689. doi: 10.1038/s41598-025-96657-5 (PMC11993596; doi:10.1038/s41598-025-96657-5)
Supplement: Supplementary file 1 — Supplementary Material 1 [file 41598_2025_96657_MOESM1_ESM.docx]

Supplemental Fig1:


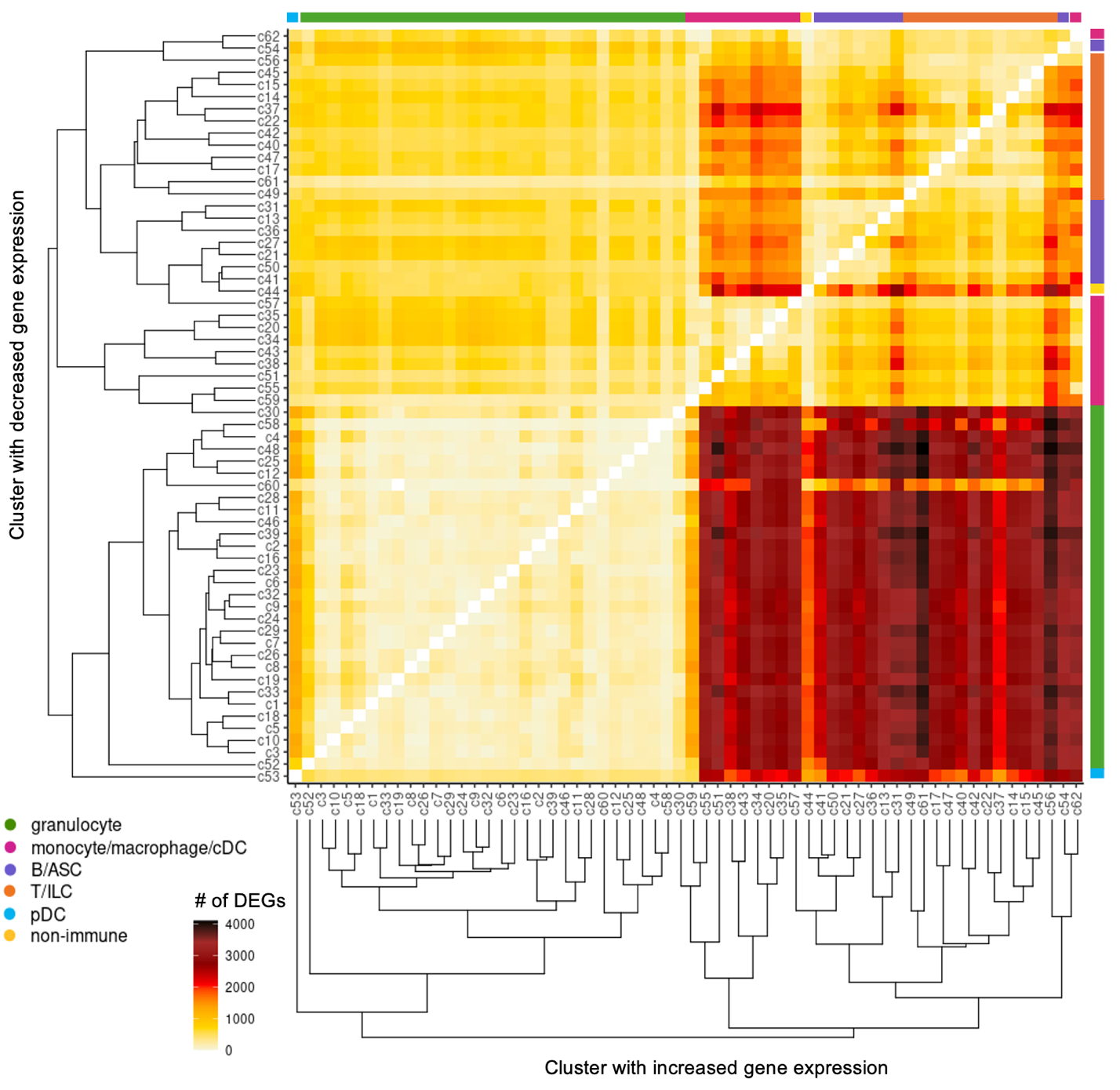


**Supplementary Figure 1. Transcriptional heterogeneity of clusters captured via pairwise differential gene expression analysis.** Heatmap depicting the number of DEGs for each pairwise comparison of clusters captured via scRNA-seq. Fill color within the heatmap indicates the number of DEGs for each comparison. In the absence of any recovered DEGs, fill color is white. Clusters with increased gene expression in a pairwise comparison are shown on the x-axis. Clusters with decreased gene expression in a pairwise comparison are shown on the y-axis. Clusters are ordered on axes based on hierarchical ordering of clusters shown with identical trees on the left and bottom of the heatmap. Color bars on the top and right of the heatmap indicate cell type assignment of clusters. DEGs were identified as having expression in at least 10% of cells in one cluster being compared, having a logFC > 0.25, and having a corrected p-value < 0.05. >4.3 million DEGs were recovered from all pairwise comparisons and are available in a repository at Ag Data Commons, as detailed in the Data Availability statement.

Abbreviations: ASC (antibody-secreting cell); cDC (conventional dendritic cell); DEG (differentially expressed gene); ILC (innate lymphoid cell); logFC (log fold change); pDC (plasmacytoid dendritic cell); scRNA-seq (single-cell RNA sequencing)

Supplemental Fig 2


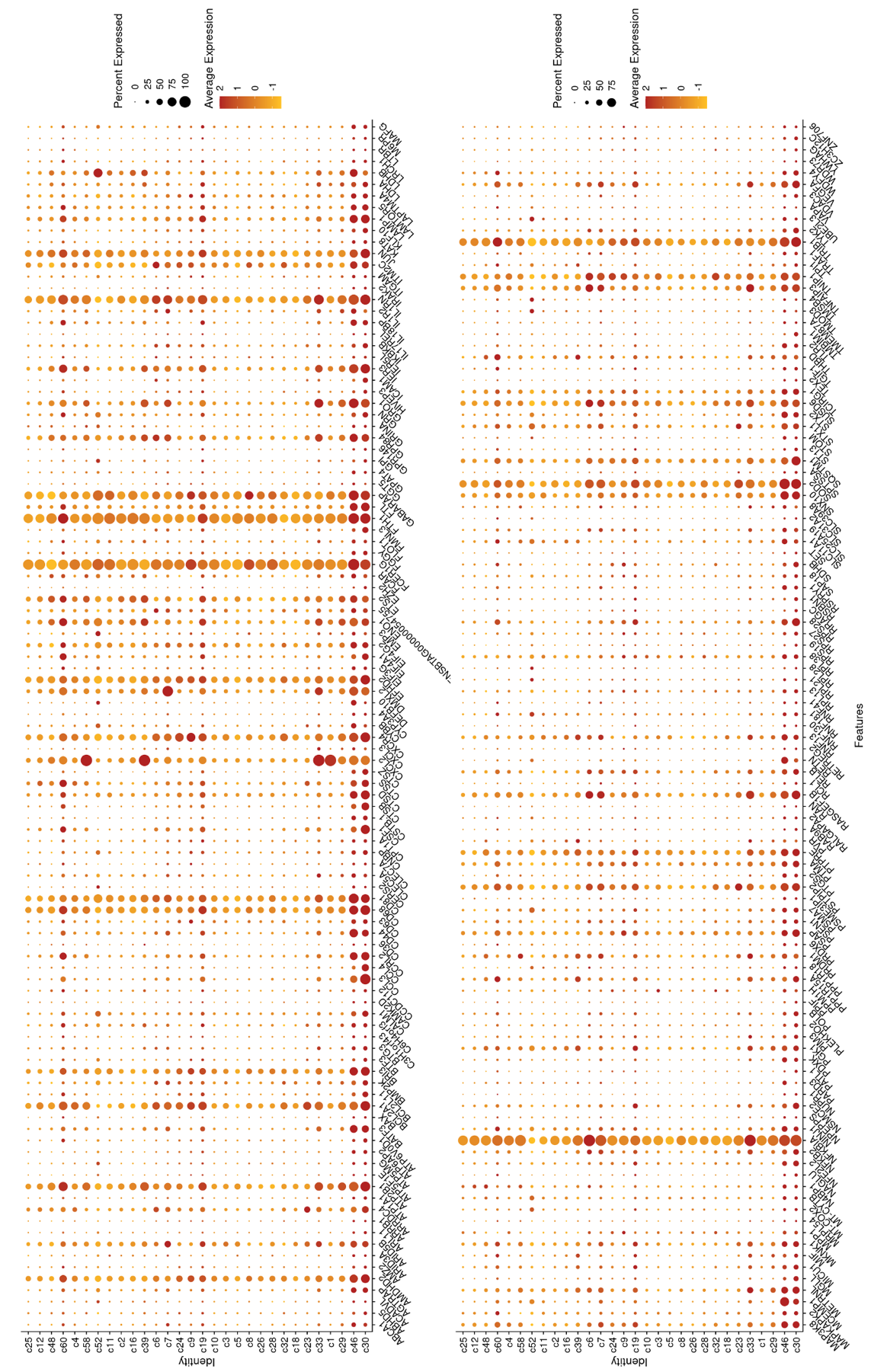


**Supplementary Figure 2.** Dot plot of signature genes (x-axes) and their expression patterns across granulocyte clusters (y-axes). Signature genes were identified for node 1 shown in **Figure 4A**. Dot size within the plot indicates the percentage of cells in a cluster expressing a gene. Dot fill color indicates the relative expression level of a gene in a cluster. The phylogenetic tree on the left shows relatedness of granulocyte clusters, identical to as shown in **A.**
